# Scoping Review of Chatbot‐Based Approaches to Cancer Patient Education

**DOI:** 10.1155/nrp/2147056

**Published:** 2026-05-05

**Authors:** Tahere Talebi Azadboni, Meysam Rahmani, Haleh Ayatollahi

**Affiliations:** ^1^ Department of Health Information Management, Saveh University of Medical Sciences, Saveh, Iran; ^2^ Health Management and Economics Research Center, Health Management Research Institute, Iran University of Medical Sciences, Tehran, Iran, iums.ac.ir

**Keywords:** cancer, chatbot, patient education

## Abstract

**Introduction:**

Patient education with interactive tools such as chatbots plays a critical role in improving health outcomes. As a number of studies examined the application of chatbots for educating cancer patients, this study aimed to review different aspects of using this technology in cancer patient education.

**Materials and Methods:**

A scoping review was conducted using the PRISMA‐ScR framework to investigate different applications of chatbots for cancer patient education. Relevant publications were found between 2014 and 2023 via thorough searches in PubMed, Web of Science, Scopus, IEEE Xplore, ACM Digital Library, ProQuest, and the Cochrane Library. The screening process was completed using a set of inclusion and exclusion criteria. Then, relevant data were extracted, and the results were summarized in tables and reported descriptively.

**Results:**

Overall, 10 studies met the inclusion criteria. These studies examined the use of chatbots for cancer patient education. Seven studies evaluated the design and usability of and user attitudes towards chatbots, while three studies investigated the accuracy and reliability of chatbot responses. Chatbots were available via mobile apps (*n* = 4), web‐based tools (*n* = 2), desktop applications (*n* = 2), and messaging platforms (*n* = 3), and some studies provided more than one communication channel. Reported clinical impacts included a decrease in mortality rates and enhanced healthcare outcomes and quality of life for patients, while nonclinical impacts encompassed a decrease in workload for care providers and providing them with the required information. Some challenges included technical limitations, insufficient digital literacy, limited accessibility, and the inability to replace human interactions.

**Conclusion:**

This study provided an overview of chatbot applications in cancer patient education. Future research should focus on identifying practical strategies for chatbot design and implementation, along with rigorous evaluations of their clinical and nonclinical effectiveness in cancer patient education.

**Implications for the Profession and/or Patient Care:**

This study contributed to identifying applications and challenges of using chatbots in cancer patient education. The results can help to improve the development of chatbots, especially for educating patients with cancer.

**Reporting Method**
 We have adhered to relevant EQUATOR guidelines, in particular to the PRISMA‐ScR checklist.

## 1. Introduction

Patient education is a process that allows individuals to make informed choices about health‐related behaviors and to enhance health outcomes by promoting compliance with medical treatment plans and healthy lifestyles. Nevertheless, changing behavior is a sophisticated process that demands more than the acquisition of knowledge. Patient education is now a firmly established and indispensable part of healthcare. Patients who are educated about their conditions are more likely to be aware of how to manage their own medical and healthcare requirements. Patient education has also been described as a primary facet of quality cancer care. It has been revealed that educating patients with cancer can decrease hospital readmission and emergency department visits and enhance the quality of life of patients and caregivers [1]. During cancer care, patients can be provided with different types of treatments, such as chemotherapy and radiotherapy. Side effects of chemotherapy are extremely common in patients with cancer, who frequently have to self‐manage their symptoms at home. However, nonadherence to treatment due to chemotherapy‐related side effects can result in decreased survival [2]. In addition, increased utilization of oral chemotherapy, the shift of treatment responsibility from caregivers to patients, the escalation in the number of cancer patients, and the chronicity of cancer have led to the majority of cancer patients receiving treatment at home. Because of this, patients and their families have to be aware of self‐management practices in chronic conditions [3].

Currently, the website of most hospitals and cancer treatment centers provides information regarding cancer care. But this information is typically provided as a list of topics that is neither interactive nor tailored to the individuals. Patients with cancer, their families, and other people who seek information typically confront difficult and stressful circumstances. In fact, they look for accurate information that is relevant to their particular circumstances [4]. Patient education has traditionally been provided through face‐to‐face individual instruction by health care providers, group classes, and written materials like brochures. Yet these methods ask patients and their families to pay attention to large amounts of new information when they may be feeling unwell, anxious, or distressed, which is contrary to fundamental principles of effective learning. Furthermore, due to limited health literacy, individuals typically lack adequate motivation to comply with the educational material presented by the healthcare professionals. Face‐to‐face education is also problematic due to costs relating to taking time off work, transportation costs, and discomfort or aversion related to group learning settings [5, 6]. In addition, limited access to educational resources may increase disparities for patients with lower socioeconomic status or those living in geographically remote locations distant from treatment facilities [5, 7].

To overcome these difficulties, the use of information technology has eased communication between healthcare professionals and patients. The field of digital health includes a wide range of technologies such as mobile health, health information technology, wearable technology, telehealth, telemedicine, personalized medicine, and text messaging. These technologies provide healthcare services irrespective of distance, location, or time [8]. Patient education is also provided through standardized templates, such as written messages and various multimedia tools, such as videos, audio, and interactive games [8]. Among these technologies, the use of conversational systems, particularly chatbots, has expanded considerably. Chatbots, also known as virtual assistants, conversational agents, educational agents, intelligent tutoring systems, and smart assistants, are software applications that simulate text or voice conversations and interact with users on specific topics in a natural, conversational manner using textual or auditory input [9].

Today, the application of artificial intelligence (AI), machine learning, and deep learning techniques has enabled the development of advanced chatbots that can be meaningfully integrated into medical education and healthcare. Investments in intelligent approaches and advanced technologies for personal health education are now more necessary than ever. Furthermore, integrating digital technology into teaching practices has become a global imperative [10]. Chatbots can be considered a cost‐effective technology solution that can enrich personalized learning environments and create customized content. These systems have inherent scalability, thus benefiting multiple users at once by solving particular difficulties, offering solutions to queries, and encouraging personalization. Some applications of this technology include gathering and delivering information, generating and responding to inquiries, collecting end‐user feedback, and providing personalized health and medical information to patients via mobile and web‐based platforms. Additionally, the ease of use of chatbots enables instant responses to users [11]. Given the potential to replace traditional conversational education methods and save workforce resources, chatbots have attracted growing attention in the field of patient education [12].

The role of chatbots in cancer patient education has been explored in a few studies [11–15]. For instance, in head‐and‐neck oncology, Kuşcu et al. studied the accuracy and reliability of ChatGPT’s responses [15]. Görtz et al. investigated other examples of automated assistance in patient care, including the work of Pan et al., who assessed chatbot responses to cancer‐related questions [11]. Although there have been several review papers on the use of chatbots in oncology or cancer education, most have either targeted one particular type of cancer (such as breast cancer) or focused on very specific aspects of chatbots, such as usability, patient satisfaction, and technical features. For example, Lin et al. conducted a systematic review and meta‐analysis on chatbots for breast cancer education and reported a high level of patient satisfaction and improved knowledge acquisition, but their aim was limited to a single cancer type [13]. Xu’s review provided insight into chatbot applications across various healthcare and oncology domains (including diagnosis, treatment, and workflow efficiency) but did not focus on the nature and educational impacts for cancer patients [16]. Wang’s narrative review synthesized chatbot applications within oncological care broadly and did not have a dedicated synthesis of its educational impact [17]. Similarly, Li and Wu’s scoping review focused on the usability and experiences of patients with conversational agents, without comprehensive coverage of design, deployment platforms, and clinical impacts [18]. In fact, there is a considerable body of evidence that needs to be synthesized in order to understand how and where chatbots can be utilized in cancer patient education, and it seems that no review has yet systematically mapped these dimensions collectively across different cancer types. A comprehensive synthesis is therefore needed to clarify inconsistent evidence, identify underexplored challenges, and provide an integrated understanding of how chatbots function as educational tools in oncology, covering clinical and nonclinical impacts, as well as challenges and limitations. This approach can also enhance the design of chatbots by making them more precise, useful, scientifically valid, and dependable while reducing restrictions. Moreover, it would ease the acceptance and integration of the devices into clinical workflows by caregivers, patients, and their families. Therefore, the current study aimed to review different aspects of using chatbots for cancer patient education. The results can help to identify limitations of the existing interventions and opportunities for optimizing chatbot design and implementation.

## 2. Methods

This scoping review was conducted in accordance with the Preferred Reporting Items for Systematic Reviews and Meta‐analyses extensions for Scoping Reviews (PRISMA‐ScR) guideline in 2025. In this study, articles published from 2014 to 2023 were searched, and to complete the study, Arksey and O’Malley’s framework was used. Before conducting this research, ethics approval was sought from the University Ethics Committee [19].

### 2.1. Stage 1: Identifying the research question.

In this study, the research questions are as follows:

1. What are the applications of using chatbots for cancer patient education?
2. What are the chatbot features for cancer patient education?
3. What are the clinical and nonclinical impacts of using chatbots for cancer patient education?
4. What are the challenges and limitations associated with the use of chatbots in cancer patient education?

### 2.2. Stage 2: Identifying Relevant Studies

To identify relevant studies, comprehensive searches were performed in PubMed, Web of Science, Scopus, IEEE Xplore, ACM Digital Library, ProQuest, and the Cochrane Library from 1^st^ January 2014 to 31^st^ December 2023. Searches used relevant keywords that were combined using Boolean operators AND and OR. Standard keywords were also selected using Medical Subject Headings (MeSH). The detailed search strategy for each database is provided in the supporting file (here).

### 2.3. Stage 3: Study Selection

This review included all original studies published in English between 1^st^ January 2014 and 31^st^ December 2023 that discussed the use of chatbots for cancer patient education. The chatbot was defined as a conversational agent that uses techniques of AI, such as machine learning, natural language processing, or rule‐based adaptive algorithms, to generate responses or to personalize interactions. Studies that discussed chatbots with no AI components, like simple scripted or static question‐answer systems, were excluded. The included studies had to be empirical studies published in a peer‐reviewed journal or an international conference proceeding. Inaccessible full texts, review articles, editorials, letters to the editor, short reports, and conference abstracts were also excluded. In addition, studies were excluded if they did not evaluate the chatbot intervention in any form, such as usability, accuracy, or clinical and nonclinical impacts. The retrieved articles were organized using the EndNote software, and after removing duplicates, the remaining articles were screened in terms of the title and abstract relevance to the aim of the study. Then, the full text of eligible studies was retrieved and reviewed. TTA and HA contributed to screening the articles independently, and any disagreements between them were resolved by discussing the issue with the third author (MRK).

### 2.4. Stage 4: Charting the Data

Data extraction was performed by using a data collection form with fields for the name of the author(s), year of publication, country, objectives of the study, research methodology, type of cancer, application of the chatbot, chatbot features, clinical and nonclinical impacts of chatbots, as well as limitations and challenges of chatbots. The data extraction form was designed using Microsoft Excel 2020.

### 2.5. Stage 5: Collating, Summarizing, and Reporting the Results

The method of content analysis was used to extract data. Then, the extracted data were tabulated, summarized, and reported descriptively.

## 3. Results

**FIGURE 1:**
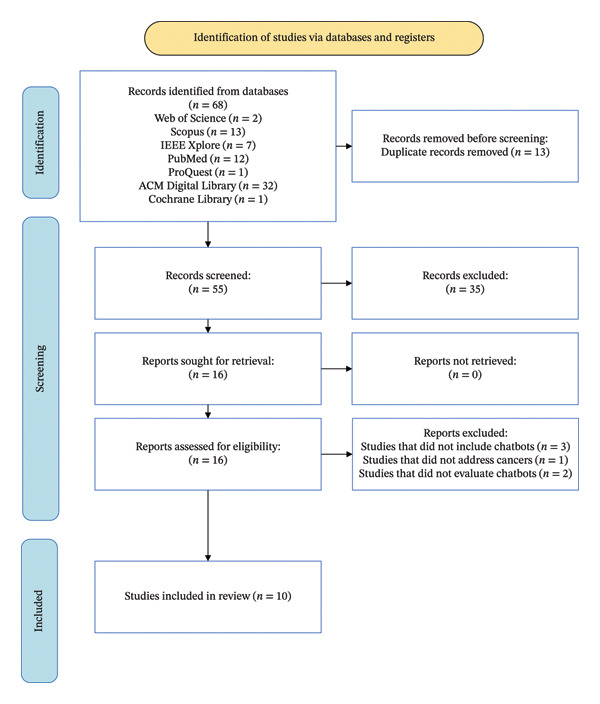
Process of selecting articles.

A total of 68 studies were initially identified through database searches. After eliminating duplicates, 55 articles remained. These articles were screened in terms of title and abstract, and irrelevant studies were removed. Afterward, the full texts of the remaining articles were examined to determine their eligibility. Ultimately, the full texts of 16 articles were reviewed. Among these, three articles were excluded, as they used chatbots in which AI was not used (excluded studies did not fit the definition of AI‐based chatbots, just used static scripts, and did not include machine learning or natural language processing), one study was excluded for not focusing on cancer, and two studies were removed because they did not include any evaluation of the chatbot intervention. Finally, 10 articles were selected to be included in the review study. This selection procedure was conducted based on the PRISMA‐ScR guideline (Figure 1).

### 3.1. Characteristics of the Selected Studies

As Table 1 shows, the highest frequency of publications was in 2023 (*n* = 5). Geographically, 50% of the studies were conducted in the United States. In most articles (*n* = 7), the research objective was to design and evaluate usability, feasibility, and user attitudes [7, 11, 12, 20–23]. The remaining three studies focused on the evaluation of the accuracy and reliability of responses given by AI‐based chatbots on cancer‐related issues [11, 15, 24]. The research methodologies included qualitative approaches [7, 20, 21], quantitative methods [11, 15, 22–24], and mixed methods [12]. The range of sample size was two to 150 participants [15, 24, 25]. One of the selected studies evaluated using a chatbot through a randomized controlled trial [24]. Seven studies recruited patients as their sample [7, 11, 20–24], and clinicians and researchers were the participants of other research studies [12, 15, 25].

**TABLE 1:** Summary of the selected studies.

No.	Author/country/year	Research objective	Research method	Cancer type/chatbot application	Chatbot features	–Clinical impact	Nonclinical impact	Challenges and limitations
1	Wu et al./USA/2014 [20]	To develop a culturally tailored educational program on cervical cancer to increase screening rates among Hispanic women in a rural farming community.	Qualitative study: Repeated interviews with the target population	Cervical cancer/cancer education	VPE was a computer character that simulated face‐to‐face conversation and acted as a patient navigator.	Decreasing the rate of cervical cancer deaths in the Latino community	Assists in simplifying the aided to close the gap in understanding the osteoporosis screening knowledge between Hispanic women and the rest of the world. The integration of cultural features enhanced overall understanding and trust in health information.	Not reported.
2	Mendu et al./USA/2018 [21]	To develop and evaluate an interactive virtual patient coach to educate and counsel Hispanic women on cervical cancer and HPV	Qualitative study/iterative design methodology; usability and pilot testing	Cervical cancer/Education and counseling	The system included 51 unique dialog statements across three assessment modules, and allowed patients to skip familiar or irrelevant information and tests with yes/no questions.	Not reported.	Potential to enhance health education.	Ambiguity about HPV and HIV contents Technical issues, such as the rapid disappearance of cell change charts and low audio volume during conversation.
3	Chetlen et al./USA/2019 [22]	To develop and deploy a chatbot for patients undergoing breast biopsy to provide evidence‐based answers to common questions	Quantitative study/A survey study to assess overall attitude	Breast cancer/education on breast biopsy	The chatbot utilizes a decision tree structure, allowing for limited initial questions on lifestyle, biopsy method, side effects, results, and exercises. The UI was designed to resemble a messaging app.	Large‐scale implementation improved healthcare knowledge, patient outcomes, and quality of life. It provided initial advice and directed users to clinical care lines.	Improved patient experience by providing healthcare information. Facilitated patients’ understanding of future methods, regardless of their technical skills, Positively influenced perceived care quality.	Not reported.
4	Piau et al./France/2019 [7]	To evaluate the feasibility and acceptance of a smartphone chatbot and its effects on the health system	Qualitative study/feasibility was tested on a patient sample using a questionnaire	All types of cancers among the elderly patients/Optimal cancer monitoring	The Infinity chatbot app was a semiautomated messaging system on smartphones that met the HIPAA data security standards. It sent sequential, short questions to track clinical symptoms, assigned scores, and sent reminders and follow‐ups.	Clinical management and ethical support of patients	Optimized nurse phone calls through chatbot data collection	Challenges included low level of technical literacy and acceptance of e‐health solutions among elderly patients.
5	Katoka et al./Japan/2021 [12]	To develop and evaluate the feasibility of a chatbot to enhance symptom management knowledge among patients with lung cancer and their caregivers in Japan.	Mixed‐methods study/developed a chatbot, then evaluated it via a web‐based questionnaire.	Lung cancer/self‐management	Chatbot was developed based on FAQs extracted from surveys, integrated into the LINE social network; used Google Cloud Dialog Flow and natural language processing to match questions and provide appropriate responses, including Japanese‐English translation for unmatched queries.	Not reported.	Medical staff could use chatbot categories to educate patients on symptom management.	A low response rate indicated potential acceptance issues; some questions had mismatched responses; the patient complained about inadequate answers. Further studies are needed to enhance patient‐chatbot interaction.
6	Görtz et al./Germany/2023 [11]	To develop and evaluate a user‐friendly medical chatbot (PROSCA) providing early prostate cancer diagnosis information to patients.	Quantitative study/developed chatbot was evaluated by 10 patients for usability, perceived benefits, and potential for improvements	Prostate cancer/education on prostate diseases, diagnostic tests, stages, and treatment options.	Chatbot designed to provide anatomical and functional information on prostate diseases, inform early diagnosis opportunities, guide diagnostic exams, and present treatment options with side effects based on tumor stage.	Assisted in early prostate cancer diagnosis.	Compared to medical brochures and websites, chatbot information was more user‐friendly, focused on addressing patient questions, and tailored to specific patient use cases. Additional information was useful for increasing patient knowledge. Repetitive tasks were reduced for physicians, allowing better personalized care.	Not reported.
7	Kuşcu et al./Turkey/2023 [15]	To evaluate the accuracy and reliability of ChatGPT’s responses to questions about head and neck cancer	Quantitative study/In total, 154 questions were collected from professional communities, institutions, patient support groups, social media, and ChatGPT responses, evaluated by two head and neck surgeons for accuracy and reproducibility.	Head and neck cancer/answering cancer‐related questions	ChatGPT was an AI model designed to generate human‐like conversational responses based on extensive data, including websites, books, and articles.	Not reported.	It was a helpful information source for patients and healthcare professionals, and supported clinical decision‐making by providing up‐to‐date information to physicians.	Reliability depended on the quality of training data. The latest version of ChatGPT is accessible only via a paid subscription, limiting public access to more accurate information.
8	Pan et al./USA/2023 [25]	To describe the quality and accuracy of information provided by four AI chatbots (ChatGPT, Perplexity, ChatSonic, Bing) on skin, lung, breast, colorectal, and prostate cancers.	Quantitative study/Cross‐sectional study comparing AI chatbot text responses to five FAQs on five common cancers using validated tools; queries extracted from Google Trends; identical prompts used for all chatbots.	Skin, lung, breast, colorectal, prostate cancers/Answering cancer questions	ChatGPT 3.5 (OpenAI), Perplexity (Perplexity.AI), ChatSonic (Writesonic), Bing AI (Microsoft).	Not reported.	Provided accurate and reliable cancer information	Responses were at an academic level, but the performance was poor. Usefulness was limited by poor readability and a lack of visual aids.
9	Tawfik et al./Egypt/2023 [24]	To evaluate and compare the effects of ChemoFreeBot on self‐care behaviors and frequency, severity, and discomfort of chemotherapy side effects in women with breast cancer.	Quantitative study/150 breast cancer women randomly assigned to ChemoFreeBot group, nurse‐led education, or usual care; measured self‐care behaviors and side effects before and after intervention; they also assessed ChemoFreeBot usability.	Breast cancer/self‐care after chemotherapy	ChemoFreeBot was designed to be compatible with the Android platform, making it widely accessible across a variety of devices. A knowledge base was created to answer natural language questions, and a conversation layer was added via the cloud API QnA Maker. Alternative questions were also provided to increase match success.	ChemoFreeBot was a functional and enhanced self‐care behaviors and mitigated chemotherapy side effects through personalized education and access to high‐quality information. Empowered nurses to educate women with breast cancer and enabled active symptom management by women.	Reduced time, physical, and financial burden for patients and was cost‐effective	Effective for basic information and simple questions, but unable to handle complex issues or understand human emotions; unlikely to replace human interaction.
10	Visvanathan/USA/2023 [23]	To evaluate an AI conversational agent providing pretest genetic education to cancer patients.	Quantitative study/AI conversational program developed on the HealthFAX platform for appropriate genetic education and patient responses were evaluated for acceptance, usage, and experience.	All cancer types/Providing genetic education	Chatbot built on the HealthFAX platform with an admin portal and virtual assistant tool, including provider referral information, introduction to genetic services, general genetics education, cancer genetics information, family history, test panel information, sample collection details, costs, possible results, and referral information. It also collected personal and family cancer history.	Not reported.	Cost‐effective. Provided valuable information to patients.	Chatbot could not be integrated into patients’ electronic health records.

*Note:* API QnA = Question and Answer Maker API.

Abbreviations: AI = artificial intelligence, ChatGPT = Chat Generative Pretrained Transformer, FAQs = Frequently Asked Questions, HIPAA = Health Insurance Portability and Accountability Act, HIV = Human Immunodeficiency Virus, HPV = Human Papillomavirus, UI = User Interface, VPE = Virtual Patient Educator.

### 3.2. Applications of Chatbots in Cancer Patient Education

According to the results, two papers focused on using chatbots for cancer patient education regardless of the type of cancer [7, 23], and other studies focused on using chatbots for a specific type of cancer, like prostate [11], breast [22, 24], cervical [20, 21], lung [12], and head and neck cancer [15]. In two studies, chatbots were used to assist self‐care strategies [12, 24], and one study helped with patient health monitoring [7]. Other studies used chatbots to give more information about breast biopsy [22], cancer genetic counseling [23], general education, consultations, and responding to patient inquiries [11, 15, 24].

### 3.3. Methods for Developing Chatbots in Cancer Patient Education

The results showed that chatbots designed for general education were mainly based on OpenAI’s ChatGPT, Perplexity, Writesonic’s ChatSonic, and Microsoft’s Bing chatbot.

Two chatbots included animated characters and could engage with users in vivo [20, 21]. Most chatbots were constructed from FAQs, which resulted in a portfolio of answers to a limited range of cancer‐specific questions [7, 12, 22, 23]. In two studies, more comprehensive chatbots were created using deep learning techniques and knowledge base construction [11, 24].

Moreover, four chatbots were implemented as mobile applications [7, 12, 22, 24], two were web‐based [11, 23], and two were desktop applications [20, 21]. To facilitate more effective communication, three studies used their educational chatbots as messaging services [7, 12, 22]. Specifically, in Kataoka’s study, the chatbot was integrated within the LINE messaging platform, and in Visvanathan’s study, the chatbot was embedded in the HealthFAX platform [12, 23].

### 3.4. Impact of Using Chatbots on Cancer Patient Education

The application of chatbots in cancer education has both clinical and nonclinical impacts. While many studies found no clinical impacts [11, 12, 15, 21, 23], some researchers reported that lowering mortality rates [20], enhancing healthcare outcomes and patients’ quality of life [22], applying appropriate treatment strategies [22], and treatment management [7], aiding in early cancer detection [11], reducing chemotherapy side effects and enhancing symptom control [24] were among the clinical impacts.

The nonclinical impacts of using chatbots for cancer patient education included the ease of health data collection [20], overcoming cultural and language barriers to obtain knowledge [20], ease of health and hygiene education [11, 12, 21, 22], improved communication between patients and healthcare providers [7], decreased workload for care providers, time‐saving [11], provision of information to medical and therapeutic experts [15], assistance with clinical decision‐making [15], accurate and reliable information delivery to patients [11], and a reduction of the temporal, physical, and financial burdens on patients [23, 24]. The results of the evaluation studies about the use of chatbots were encouraging and satisfactory. No study identified dissatisfaction or deterioration of patients’ complaints after using chatbots. Moreover, all studies emphasized that chatbots are useful, supportive tools.

### 3.5. Challenges and Limitations of Using Chatbots for Cancer Patient Education

As Table 1 shows, challenges, and limitations related to the use of chatbots for educating cancer patients could be grouped as follows.

#### 3.5.1. Technical Challenges

A number of technical challenges were noted in the included studies. Mendu et al. identified usability issues, such as rapidly disappearing visual charts and poor audio volume, which interfered with users’ capability to follow educational content during interactions [21]. Similarly, Pan et al. noted that while AI chatbots generally exchanged information correctly, their usability was limited by poor readability and the absence of visual aids, which reduced accessibility for people with different levels of health literacy [25]. In addition, Visvanathan et al. reported the difficulty of integrating the chatbot used in genetic education with electronic health records and its inability to support personalized recommendations and automated documentation [23]. However, inconsistencies in some questions from the patients and the responses from the chatbot were noted by Kataoka et al. to be indicative of weaknesses in the natural language processing algorithms being used [12]. According to Tawfik et al., the chatbot did not allow for addressing complex user questions or picking up emotional nuances, which again reflected limitations in AI capability [24].

#### 3.5.2. Accuracy, Appropriateness, and Readability of Chatbot Responses

Several studies pointed out challenges related to the accuracy, appropriateness, and readability of the information generated by chatbots. In the study conducted by Görtz et al., there were concerns over the accuracy of the content provided by chatbots, which depended on the quality of the data sources [11]. Küşcu et al. pointed out the uncertainty about the databases and training materials used for the AI‐generated responses; thus, there is still no full confidence in that information [15].

According to Pan et al., although generally accurate, the cancer‐related information produced by several chatbots was often written at an overly academic level, which limited readability and made comprehension difficult for users without medical backgrounds [25]. Some of these tools were not fully able to answer all users’ questions, reflecting a gap in content coverage and response appropriateness.

#### 3.5.3. Low Level of Digital Literacy and Attitudes towards Technology

Some researchers showed that older cancer patients had a low level of digital literacy and acceptance of e‐health solutions. Therefore, if chatbots are developed with adequate reliability and efficiency, they may fail to achieve the intended results, such as effective use of the system, completion of core educational tasks, and improvement in patient knowledge, if users are unable to interact with them properly due to limited technical skills or negative attitudes towards technology [7]. Also, Chetlen et al. identified that patients with little prior experience using messaging apps required more support to familiarize themselves with the chatbot interface to use the educational contents [22]. These findings highlighted the importance of user‐centered design, intuitive interfaces, and targeted support or training, especially for those who need more support.

#### 3.5.4. Access and Cost‐Related Challenges of Chatbots

Several studies highlighted that cost‐related challenges were critical barriers in implementing chatbots for cancer patient education. A few chatbots, such as ChatGPT, require purchased subscriptions to ensure the accuracy and timely update of information. However, they may not be accessible to patients who do not have the financial resources to purchase such subscriptions [15]. Moreover, the use of chatbots usually requires specific devices like smartphones, tablets, or computers, along with stable internet connectivity, which may not necessarily be available to all patients [23, 24]. These issues can affect older adults, the low‐income population, and patients from rural or resource‐limited settings. Therefore, financial and technical accessibility must be addressed to ensure that the implementation of chatbots in cancer education is available to all patients equitably.

#### 3.5.5. Handling Complex Clinical Issues and Human Emotions

Various studies emphasized the intrinsic limitations of chatbots in handling complex clinical matters and interpreting human emotions. These limitations are discussed in the following sections.

Chatbots cannot process the subtle emotional context and/or complex patient queries. This was manifested in the report by Tawfik that ChemoFreeBot could only manage simple education and symptom management and was unable to answer more complex questions or assess patients’ emotional condition [24].

Several chatbots failed to include the functionality of personalization, thus limiting the degree to which responses can be tailored to individual patient needs. According to Visvanathan et al., the AI conversational agent was able to provide genetic education but could not tailor interactions to specific emotional or psychosocial concerns [23].

Although many studies demonstrated the usefulness of chatbots at an informative level, empirical evidence supporting the effectiveness of chatbots in providing emotional support or handling highly individualized scenarios remains limited [23, 25].

These findings suggested that chatbots should ideally be treated as adjunctive technologies, augmenting rather than replacing human healthcare providers, especially when complex medical decisions or empathetic patient care come into play.

### 3.6. Synthesis

The results showed that the use of chatbots in the education of cancer patients is a new and developing approach that has been used in various cancers such as breast, prostate, cervix, lung, and skin and is mainly aimed at promoting awareness, improving self‐care behaviors, and increasing patient communication with the healthcare team [7, 12, 24]. Despite capabilities such as accessibility, time and cost saving, and relative importance of content personalization, challenges such as low accuracy and legibility of information, weaknesses in responding to complex clinical questions, and the lack of understanding of human emotions have been reported [11, 23–25]. The limitation of digital literacy in the elderly, the lack of technological infrastructure, and the cost of sharing some services are also access barriers [7, 15, 22–25]. Also, technical problems such as difficulty in integration with EHRs and inefficient natural language processing algorithms are among the other prominent challenges [11, 12, 25]. In sum, chatbots should be considered as supporting and complementary means of nursing care that, through user‐centered design, a justice‐based approach, and continuous evaluation, can play an effective role in teaching and empowerment of cancer patients.

## 4. Discussion

This scoping review synthesized current evidence on chatbots for educating cancer patients, highlighting both their potential and limitations. Overall, chatbots have shown promise as adjunctive tools in offering personalized information, supporting self‐management, and facilitating communication between patients and healthcare providers. However, a health chatbot needs to be used by a variety of users over diverse treatments and diagnoses, and it has to be fed large amounts of data, such as medical terminologies, symptoms, and treatments. The accuracy of these data is of paramount importance, as unchecked misinformation is potentially dangerous for the patients [26]. Wang et al. pointed out that with the complexity of AI technology, it is necessary to conduct extensive user trials and iterations in order to maximize user satisfaction and adoption before large‐scale deployment. In the future, chatbots will have more access to richer datasets to support their knowledge pools and expand to be broader [17].

While previous reviews paid attention to specific types of cancers, such as Lin et al., who focused on using chatbots in breast cancer [13], the current review synthesized evidence more broadly and allowed gaining deeper insights into the benefits and limitations of chatbots in cancer patient education. In terms of usage, these chatbots not only facilitate the educational content and self‐care guidance but also provide personalized interactions, such as recalling the medication and reducing the symptoms, as a helpful tool for long‐term management of the disease [2, 3, 7, 11]. The development approach of these systems is generally based on collaborative design frameworks with patients and health professionals, as emphasized in studies such as Xu et al., on the necessity of extensive usability tests and performing successive reforms to improve usability [16]. In terms of clinical effectiveness, the evidence shows that the chatbots can increase the knowledge of patients and improve adherence to treatment. Meanwhile, nonclinical effects such as the reduction of workload of healthcare providers and increased access to information outside work hours were also mentioned in the study [13]. However, as Lin et al. and Xu et al. have pointed out, the friendly user interface and the level of interaction created by the chatbots are among their key strengths that increase the acceptance and satisfaction of users [13, 16].

Apart from clinical and nonclinical impacts, the use of chatbots may have several challenges and limitations. For example, while the usability issues were emphasized in the past studies, the accuracy and readability of the chatbot content should not be underestimated. Some studies showed that chatbots can provide clinically correct information, but sometimes their responses were highly academic, lacking visual aids, or mismatched with user queries [3, 4, 25]. Such limitations raise questions about content quality, clarity, and alignment with the health literacy level of patients [7]. Second, while most past reviews assumed that chatbots could perform well in supporting patient education, our synthesis showed that chatbots have a limited capacity in handling complex clinical inquiries and even interpreting human emotions. Included studies in this review proved that while chatbots have poor performance in subtle patient concerns or personalization to emotional/psychosocial contexts, their responses are still quite incapable of being empathetic [24]. This contrasts with the general claim of efficiency from previous reviews and pinpoints treating them as adjunctive rather than replacement tools for human health service providers [27].

Third, various access levels and equity issues were rarely discussed in the previous reviews. Our results showed that financial barriers and subscription fees for AI chatbots like ChatGPT, technological preconditions—notably, smartphones and stable internet connections—and limitations in digital literacy are likely to affect older adults, low‐income individuals, and patients in rural areas, particularly. These findings underscore the need to develop accessible, inclusive chatbot interventions and to invest in sufficient user support and training [7, 15, 22–24].

Finally, technical integration remains a notable challenge. While past reviews focused more on usability and engagement issues, we found specific barriers relating to a lack of interoperability with electronic health records and limitations in natural language processing, which influenced the relevance of content and user interaction. Such issues should be addressed if the clinical utility of chatbots for cancer education is to be improved and their widespread use scaled up [12, 23].

### 4.1. Research Implication

Based on the findings, several actionable directions would enhance chatbot interventions for cancer patient education. First, the use of multimodal content (e.g., text, audio, and visual) could improve comprehension and engagement. Second, interoperability with EHRs should be enhanced to support personalized education, enable automated documentation of educational interactions, and better integrate education into broader clinical workflows. Third, user‐centered optimization of interface design, including intuitive design and targeted training among less‐literate populations, will improve usability and acceptance. Fourth, content simplification may be required to ensure that it matches patients’ levels of comprehension while ensuring clinical accuracy to maximize the potential for educational effect and minimize the risk of misunderstanding complex information. Fifth, reducing cost and access barriers through strategies such as minimizing subscription requirements or ensuring compatibility with widely available devices could enhance equity of implementation across diverse socioeconomic and geographic populations. Finally, rigorous empirical examination of educational and psychosocial outcomes is still needed; much existing research has emphasized either usability or satisfaction with relatively less evaluation of learning effects, behavior change, or psychosocial impact.

### 4.2. Research Limitations

Although current findings provided some new insights about using chatbots for educating cancer patients, there were some limitations. Despite the extensive searches in various databases, some eligible studies might be missed, especially non‐English publications or unpublished papers. Furthermore, because of the heterogeneity in chatbot functionalities, educational content, and outcome measures across included studies, meta‐analysis was not possible. Nevertheless, synthesizing the evidence related to the clinical and nonclinical impacts, as well as various design features and challenges of implementation, provided a broader and more integrated perspective on the use of chatbots for cancer patient education.

## 5. Conclusions

This study highlighted the applications, development methods, and challenges associated with using chatbots in cancer patient education. The results demonstrated that overall, there is a positive perspective regarding the use of this technology. Chatbots have the potential to reduce costs, improve communication between healthcare providers and patients, deliver timely information to patients, assist medical decision‐making through the provision of up‐to‐date knowledge, and save healthcare professionals’ time. However, some challenges, such as technical challenges, accuracy, appropriateness, and readability of chatbot responses, low level of digital literacy of users and attitudes towards technology, and access and cost‐related challenges of chatbots, should not be underestimated. The findings of this research can contribute to the development of future chatbots by paying more attention to the challenges and limitations. However, the clinical and nonclinical impacts of using this technology need further investigation in further research.

## Author Contributions

Conceptualization: Tahere Talebi Azadboni, Meysam Rahmani, and Haleh Ayatollahi; methodology: Tahere Talebi Azadboni, Meysam Rahmani, and Haleh Ayatollahi; formal analysis: Tahere Talebi Azadboni and Meysam Rahmani; investigation: Tahere Talebi Azadboni and Meysam Rahmani; writing–original draft: Tahere Talebi Azadboni and Meysam Rahmani; writing–review and editing: Tahere Talebi Azadboni and Haleh Ayatollahi; supervision: Haleh Ayatollahi.

## Funding

This research was funded and supported by the Health Management and Economics Research Center, Health Management Research Institute, Iran University of Medical Sciences, Tehran, Iran.

## Disclosure

All authors have read and agreed to publish the manuscript.

## Ethics Statement

This study was performed in line with the principles of the Declaration of Helsinki. Approval was granted by the Ethics Committee of Iran University of Medical Sciences (IR.IUMS.REC.1403.359).

## Conflicts of Interest

The authors declare no conflicts of interest.

## Supporting Information

Additional material related to this study is provided as a Supporting file (Appendix A: Search strategies).

## Supporting information


**Supporting Information** Additional supporting information can be found online in the Supporting Information section.

## Data Availability

The data that support the findings of this study are available from the corresponding authors upon reasonable request.
