# Supplementary material for: Scoping Review of Chatbot‐Based Approaches to Cancer Patient Education
Source: Nurs Res Pract. 2026 May 5;2026:2147056. doi: 10.1155/nrp/2147056 (PMC13141675; doi:10.1155/nrp/2147056)
Supplement: Supplementary file 1 — Supporting Information Additional supporting information can be found online in the Supporting Information section. [file NRP-2026-2147056-s001.zip › Supplementary File.docx]

**Appendix A. Search strategies**

| **Database** | **Search strategy** |
| --- | --- |
| **PubMed** | ((Chatbot [Title/Abstract] OR “Chat robot” [Title/Abstract] OR “Conversational agent” [Title/Abstract] OR “conversational AI” [Title/Abstract] OR “dialog system” [Title/Abstract] OR “Virtual agent” [Title/Abstract] OR “social robot” [Title/Abstract]) AND (Neoplasms [MeSH Terms] OR Neoplasms [Title/Abstract] OR Tumor [Title/Abstract] OR Cancer [Title/Abstract]) AND ("Patient Education as Topic"[ MeSH Terms] OR “Patient Education” [Title/Abstract] OR “Patient training” [Title/Abstract] OR “Cancer education” [Title/Abstract] OR “Patient Teaching” [Title/Abstract] OR “Patient Instruction” [Title/Abstract])) |
| **Web of Science** | ((TI=((("Chatbot" OR "Chat robot" OR "Conversational agent" OR "conversational AI" OR "dialog system" OR "Virtual agent" OR "social robot" ) AND ( "Neoplasms" OR "Tumor" OR "Cancer ") AND ("Patient Education" OR "Patient training" OR "Cancer education" OR "Patient Teaching" OR "Patient Instruction")))) OR AB=((("Chatbot" OR "Chat robot" OR "Conversational agent" OR "conversational AI" OR "dialog system" OR "Virtual agent" OR "social robot" ) AND ( "Neoplasms" OR "Tumor" OR "Cancer ") AND ("Patient Education" OR "Patient training" OR "Cancer education" OR "Patient Teaching" OR "Patient Instruction")))) OR KP=((("Chatbot" OR "Chat robot" OR "Conversational agent" OR "conversational AI" OR "dialog system" OR "Virtual agent" OR "social robot" ) AND ( "Neoplasms" OR "Tumor" OR "Cancer ") AND ("Patient Education" OR "Patient training" OR "Cancer education" OR "Patient Teaching" OR "Patient Instruction"))) |
| **Scopus** | TITLE-ABS-KEY (("Chatbot" OR "Chat robot" OR "Conversational agent" OR "conversational AI" OR "dialog system" OR "Virtual agent" OR "social robot" ) AND ( "Neoplasms" OR "Tumor" OR "Cancer ") AND ("Patient Education" OR "Patient training" OR "Cancer education" OR "Patient Teaching" OR "Patient Instruction")) |
| **IEEE Xplore** | ((“Chatbot” OR “Chat robot” OR “Conversational agent” OR “conversational AI” OR “dialog system” OR “Virtual agent” OR “social robot”) AND (“Neoplasms” OR “Tumor” OR “Cancer “) AND (“Patient Education” OR “Patient training” OR “Cancer education” OR “Patient Teaching” OR “Patient Instruction”)) |
| **ACM Digital Library** | [[All: "chatbot"] OR [All: "chat robot"] OR [All: "conversational agent"] OR [All: "conversational ai"] OR [All: "dialog system"] OR [All: "virtual agent"] OR [All: "social robot"]] AND [[All: "neoplasms"] OR [All: "tumor"] OR [All: "cancer "]] AND [[All: "patient education"] OR [All: "patient training"] OR [All: "cancer education"] OR [All: "patient teaching"] OR [All: "patient instruction"]] |
| **ProQuest** | title((("Chatbot" OR "Chat robot" OR "Conversational agent" OR "conversational AI" OR "dialog system" OR "Virtual agent" OR "social robot" ) AND ( "Neoplasms" OR "Tumor" OR "Cancer ") AND ("Patient Education" OR "Patient training" OR "Cancer education" OR "Patient Teaching" OR "Patient Instruction"))) OR abstract((("Chatbot" OR "Chat robot" OR "Conversational agent" OR "conversational AI" OR "dialog system" OR "Virtual agent" OR "social robot" ) AND ( "Neoplasms" OR "Tumor" OR "Cancer ") AND ("Patient Education" OR "Patient training" OR "Cancer education" OR "Patient Teaching" OR "Patient Instruction"))) OR mainsubject ((("Chatbot" OR "Chat robot" OR "Conversational agent" OR "conversational AI" OR "dialog system" OR "Virtual agent" OR "social robot" ) AND ( "Neoplasms" OR "Tumor" OR "Cancer ") AND ("Patient Education" OR "Patient training" OR "Cancer education" OR "Patient Teaching" OR "Patient Instruction"))) |
| **The Cochrane Library** | ("chatbot" OR "chat robot" OR "conversational agent" OR "conversational AI" OR "dialog system" OR "virtual agent" OR "social robot") AND ("neoplasm" OR "tumor" OR "cancer") AND ("patient education" OR "patient training" OR "cancer education" OR "patient teaching" OR "patient instruction") in Title, Abstract, Keyword |
